# Diversity and abundance of photosynthetic sponges in temperate Western Australia

**DOI:** 10.1186/1472-6785-9-4

**Published:** 2009-02-05

**Authors:** Marie-Louise Lemloh, Jane Fromont, Franz Brümmer, Kayley M Usher

**Affiliations:** 1 Abteilung Zoologie, Biologisches Institut, Universität Stuttgart, Pfaffenwaldring 57, 70569 Stuttgart, Germany; 2 Department of Aquatic Zoology, Western Australian Museum, Locked Bag 49, Welshpool DC, Western Australia 6986, Australia; 3 School of Plant Biology, University of Western Australia, 35 Stirling Highway, Crawley, Western Australia 6009, Australia

## Abstract

**Background:**

Photosynthetic sponges are important components of reef ecosystems around the world, but are poorly understood. It is often assumed that temperate regions have low diversity and abundance of photosynthetic sponges, but to date no studies have investigated this question. The aim of this study was to compare the percentages of photosynthetic sponges in temperate Western Australia (WA) with previously published data on tropical regions, and to determine the abundance and diversity of these associations in a range of temperate environments.

**Results:**

We sampled sponges on 5 m belt transects to determine the percentage of photosynthetic sponges and identified at least one representative of each group of symbionts using 16S rDNA sequencing together with microscopy techniques. Our results demonstrate that photosynthetic sponges are abundant in temperate WA, with an average of 63% of sponge individuals hosting high levels of photosynthetic symbionts and 11% with low to medium levels. These percentages of photosynthetic sponges are comparable to those found on tropical reefs and may have important implications for ecosystem function on temperate reefs in other areas of the world. A diverse range of symbionts sometimes occurred within a small geographic area, including the three "big" cyanobacterial clades, *Oscillatoria spongeliae*, "*Candidatus *Synechococcus spongiarum" and *Synechocystis *species, and it appears that these clades all occur in a wide range of sponges. Additionally, spongin-permeating red algae occurred in at least 7 sponge species. This study provides the first investigation of the molecular phylogeny of rhodophyte symbionts in sponges.

**Conclusion:**

Photosynthetic sponges are abundant and diverse in temperate WA, with comparable percentages of photosynthetic to non-photosynthetic sponges to tropical zones. It appears that there are three common generalist clades of cyanobacterial symbionts of sponges which occur in a wide range of sponges in a wide range of environmental conditions.

## Background

Sponges (Phylum *Porifera*) are sessile aquatic metazoans that are found in all aquatic habitats and have important roles in marine ecological processes. Fossil records dating back to the Late Cambrian era 509 million years ago show that sponges have survived largely unchanged in their general structural organization [1]. So far about 7,000 extant species are described [2]. As filter feeders, sponges filter food particles from the water pumped through their body and bacteria are the main component of the sponge diet [3,4].

Photosynthetic sponges are very diverse in terms of the taxonomy of hosts and symbionts and their biogeography, occurring around the world in tropical and temperate oceans. They occupy a similar niche to hard corals, being filter-feeding benthic primary producers that provide food and shelter for a range of reef organisms. Photosynthetic symbionts include cyanobacteria, dinoflagellates, rhodophytes, chlorophytes and diatoms [5-7]. These symbionts provide photosynthates [8-10] and possibly fixed nitrogen [11] to the sponge host. In addition to donating energy and carbon, cyanobacteria may benefit sponges by the production of secondary metabolites that act as antibacterials and deter predators [12,13].

The most common group of photosynthetic symbionts in sponges are the cyanobacteria, occurring in at least 38 sponge genera belonging to the Classes Calcarea and Demospongiae [14,15]. Despite their apparent importance to many sponges, only two sponge species (*Chondrilla australiensis *and *Diacarnus erythraenus*) have been studied to determine how they acquire their cyanobacterial symbionts. Vertical transmission, which is considered to be an indicator for mutualism [16], was demonstrated in both sponge species using transmission electron microscopy (TEM) and molecular techniques [17-19]. This passage of the symbiont from parent to offspring may benefit sponge larvae by providing photosynthetically fixed energy before they are able to feed, in addition to benefiting the sessile adult sponges. Vertical transmission via sponge larvae has also been shown for an alphaproteobacterial symbiont common to many marine sponges [20].

It is not known conclusively if sponges that vertically transmit symbionts are able to recognise them. Many sponges harbor large numbers of extracellular bacteria in their mesohyl, and if they are fed these bacteria together with food bacteria, the sponges are able to discriminate between them [21-23]. This suggests that the symbionts are either "invisible" to the sponge due to a physical capsule or molecular shield (suggestions only), or that the sponge is able to recognize them as symbionts. It is likely that different symbionts have evolved different strategies for co-existing with sponges. Recent studies using molecular techniques show that symbiotic microorganisms in sponges are phylogenetically diverse and that sponge-specific microbial communities exist that are different from that of seawater [7,15,24].

A phylogenetic analysis of sponge derived cyanobacterial 16S rDNA sequences showed that more than three-quarters fell into monophyletic, sponge-specific clusters [7]. For one cluster of symbiotic cyanobacteria the name *'Candidatus *Synechococcus spongiarum' was proposed [25] as this species has not been found in water samples and is phylogenetically distinct from its nearest free-living *Synechococcus *relative [26]. Recent research demonstrates that this is the most common cyanobacterial symbiont in sponges, occurring around the world [7]. Other important cyanobacterial symbionts are the filamentous *Oscillatoria spongeliae *and the relatively large coccoid *Synechocystis trididemni*, a symbiont also found in ascidians.

Photosynthetic sponges are common in tropical regions [27] and many of these sponges may derive a significant proportion of their nutrition from their photosynthetic symbionts [8]. Wilkinson [28] proposed that phototrophic sponges are more abundant in oligotrophic waters than in areas with high nutrient levels, and this appears to have lead to the assumption that they are largely restricted to tropical and sub-tropical zones [29]. Although photosynthetic sponges have been well documented in tropical regions to our knowledge only one study has investigated their abundance in a temperate region. Roberts and colleagues [29] estimated that over 65% of temperate reef sponges from New South Wales, Australia, may contain photosynthetic symbionts. Polar regions represent another unique environment for sponge symbioses. Webster et. al. [30] reported diatoms and dinoflagellates in Antarctic sponges, but do not report cyanobacterial symbionts. Several other studies [31-33] have also demonstrated the presence of symbiotic diatoms in Antarctic sponges.

The existence of sponge-specific microorganisms has been accepted but many gaps remain in our knowledge of the identity of the symbionts, the mechanisms that affect the microbial diversity in the sponges, and the physiology of the symbionts. In this study we use the term 'symbiosis' as the intimate association of two organisms, including mutualisms and parasitism. The aim of this study was to determine the percentages of photosynthetic sponges in a temperate region and compare this with previous studies of tropical regions, and to explore the biodiversity of photosynthetic symbionts of sponges and their relative abundance in temperate south-western Australian waters. 5 m belt transects with high numbers of sponges were selected and studied using optical and molecular techniques. Additionally, *in situ *measurements of photosynthetic activity were carried out for some sponges using a Diving PAM (pulse-amplitude-modulation) fluorometer.

## Methods

### Sponge collection and preservation

5 m belt transects were located in coastal areas of temperate southern Western Australia in depths ranging from 3 m to 8.2 m and with different ecological conditions (see Table 1). Sponge samples that were directly under the 5 m transect line or approximately 30 cm beside the line were collected. The study areas were located in the Perth metropolitan region (32°03'S, 115°45'E), Busselton Jetty (33°30'S, 115°10'E), and Eagle Bay, near Busselton (33°33.036'S, 115 °04.056'E) (Table 1. Please see Ref. [26] for map) and were investigated using SCUBA. These areas have high chlorophyll *a *levels all year indicating the presence of nutrients, and levels are particularly high in the winter months when rainfall and run-off washes fertilizers and other nutrient sources into the ocean [34]. The light level categories 'low', 'medium' and 'high' were determined at the study sites based on the presence of caves or overhangs and depth. A diving pulse-amplitude-modulated (PAM) fluorometer was used whenever possible to determine the presence of photosynthesis in sponges, and samples were only collected from sponges with a positive result. Where the PAM was not used all sponges on the transect were collected and assessed in the laboratory for photosymbionts using fluorescence microscopy. Each sample was divided into four parts and preserved in 75% ethanol, saturated DMSO solution pH 7.5 (20% DMSO, 0.25 M Na_2_EDTA pH 8, NaCl to saturation), 2.5% glutaraldehyde and -20°C on return to shore. All samples were collected with permissions and are stored at the Western Australian Museum.

**Table 1:** Transect locations: 5 m belt transects conducted in coastal areas of the Perth metropolitan region and Busselton area.

Transect label	Location	Depth	Light level	Study site
WP1	Woodman Point	3.0 m	High	West/North-West facing gently sloping wall, limestone boulders, high sedimentation rate
WP2	Woodman Point	7.0 m	High	West/North-West facing gently sloping wall, limestone boulders, high sedimentation rate
SM1	South Mole	5.7 m	Low	South facing wall with overhang
SM2	South Mole	7.8 m	Full Sun	Open sandy flat bottom
EB	Eagle Bay	7.2 m	Full Sun	Upper surface of large rock
BJ	Busselton Jetty	6.9 m	Medium	Jetty pylons
Mn*	Mindarie	8.2 m	Medium	West/South-West facing overhang

*Transect Mn was only 3 m in length.

### PAM fluorometry

An underwater pulse-amplitude-modulated (PAM) fluorometer (Diving-PAM, Walz GmbH, Germany) was used on all sponges on transects EB, BJ and Mn prior to sampling to determine which sponges were positive for photosynthesis. This allowed us to sample only sponges with photosynthetic symbionts, preventing unnecessary environmental damage and saving time. Sponges with visible surface contamination were brushed with a tooth brush before PAM readings were taken. To record rapid light curves a surface holder "DIVING-SH" was fixed on the sponge. Rapid light curves first measured the maximal YIELD in the absence of actinic light, followed by a series of 8 consecutive YIELD-measurements with increasing light intensity. The measuring intensity was 12 and the saturating light pulse intensity was 10 (width 0.8 seconds). The actinic light intensity at the beginning of the light curve measurement was 2 and the step width was 10 seconds. If the YIELD was less than 0.1 at the beginning of the measurement the sample was defined as 'negative' for photosynthetic symbionts. These sponges were noted, but not collected. As we lacked the equipment necessary to determine absorbance and reflection of light of different sponges, absolute levels of photosynthetic efficiency could not be determined.

### Light Microscopy

All samples were analyzed via fluorescence microscopy to determine the presence, distribution and density of photosynthetic symbionts. Fresh sponge samples were washed with autoclaved sea water and cross sections were cut thinly using a double-sided razor. The sections were examined by fluorescence microscopy using a Leitz Diaplan microscope (Leica Microsystems, Germany). Blue light, using a I3 filter block (450–490 nm excitation filter and 515 barrier filter), and green light, using a N2.1 filter block (515–560 nm excitation filter and 590 barrier filter), were used to detect the distribution and concentration of autofluorescent cyanobacterial or algal pigments. Cyanobacterial and rhodophyte autofluorescence appeared orange-yellow using blue light due to the presence of phycobiliproteins, and were red in colour using green light, while algae (other than rhodophytes) appeared red in blue light. All samples were categorized according to a subjective analysis of the levels of autofluorescence from photosynthetic pigments (Table 2) and the categories 'low', 'medium' and 'high' were given to describe the densities of symbionts. Samples with no autofluorescence and ambiguous samples (very low autofluorescence or potential surface contamination) were called 'negative'.

**Table 2:** Transect results: Number of individuals and species of photosynthetic sponges at different sampling sites.

Transect label	Light level	Individuals				Species			
		total	P	L-M	N	total	P	L-M	N
WP1	High Light	31	22	3	6	8	2	3	3
WP2	High Light	20	11	1	8	7	2	1	4
SM1	Low	19	15	3	1	6	2	3	1
SM2	Full Sun	11	2	6	3	5	2	2	1
Mn	Medium	20	9	3	8	11	5	1	5
EB	Full Sun	26	21	0	5	18	13	0	5
BJ	Medium	15	9	0	6	14	8	0	6

Totals:		142 (100%)	89 (63%)	16 (11%)	37 (26%)	69	34	10	25
Number of different species:						63 (100%)	30 (48%)	10 (16%)	23 (36%)

P: high levels of autofluorescence, L-M: low to medium levels of autofluorescence, N: no autofluorescence.

### Transmission Electron Microscopy

Fresh sponge biopsies (~2 mm^3^) were fixed in 2.5% EM grade glutaraldehyde in 0.05 M phosphate buffer pH 7.0 (0.05 M NaH_2_PO_4 _and 0.05 M Na_2_HPO_4_) for 3 hours then post-fixed with 1% O_s_O_4 _in 0.05 M phosphate buffer for 1 1/2 hours. The specimens were dehydrated in an ethanol series and 100% propylene oxide, then embedded in Spurr's resin [35]. Ultra-thin sections (90 – 150 nm) were cut using a Reichert ultramicrotome (Leica, Ultracut) with glass knifes. Sections were transferred to naked copper grids (75 × 300 mesh) and stained with 1% aqueous uranyl acetate for 15 minutes and lead citrate [36] in carbon dioxide free conditions for 10 minutes. The specimens were washed in double distilled water and air dried, before examination with a JEOL 2000 FX electron microscope (JOEL Ltd., Japan) operating at 80 kV.

### PCR

To avoid surface contamination the sponge samples were brushed with a toothbrush and rinsed with distilled water. Distribution of symbionts in the sponge tissue was examined via fluorescence microscopy and, where possible, about 1 mm of the sponge surface was removed with a double-sided razor to reduce surface contamination. Approximately 1 mm^3 ^pieces of sponge tissue were placed directly into the PCR reaction mixes after soaking in ddH_2_O for 5 minutes. Cyanobacterial-specific primers were used to amplify cyanobacterial DNA in sponges. The forward primer cya359F (5'-GGGGAATYTTCCGCAATGGG) [37] and the reverse primer cya1459R (5'-GGTAAYGACTTCGGGCRT) [38] were used to amplify a 1100 base pair section of the small subunit of ribosomal RNA (16S rDNA). For Denaturing Gradient Gel Electrophoresis (DGGE) a 40-nucleotide GC clamp (5'-CGCCCGCCGCGCGCGGCGGGCGGGGCGGGGGCACGGGGGG-3') was added to the forward primer cya359F and used with the reverse primer cya781 [37]. 25 μl PCR reactions contained final concentrations of 0.08 U/μl BIOTAQ DNA Polymerase (BIOLINE Australia), 1 × NH_4 _Buffer, 2 mM dNTP mix, 2.5 mM MgCl_2 _and 0.6 μM of each oligonucleotide primer. The following program was used to amplify cyanobacterial DNA: one cycle of 94°C for 4 min, 60°C for 2 min and 72°C for 2 min followed by 30 cycles of 94°C for 1 min, 60°C for 1 min and 72°C for 1 min. The program ended with 72°C for 4 min and a 4°C store. For DGGE the following PCR program was used: 95°C for 15 min, followed by 94°C for 1 min, 68°C for 1 min and 72°C for 2 min. For each subsequent cycle the temperature of annealing was reduced by 1°C until it reached 60°C, whereupon the cycle was repeated 22 times. The PCR was ended with an extension step at 72°C for 10 minutes and a 4°C hold. All PCR reactions were carried out under S1 conditions at CSIRO laboratories.

### Denaturing Gradient Gel Electrophoresis

20 μL of PCR product were added to 10 μL of loading buffer and run on a 7% polyacrylamide gel with a denaturing gradient of 20 to 60%. The wells and the top of the gel were formed with 0% urea. The gel was run for 3.5 hours at 180 V, then stained in 20 mg of ethidium bromide/ml in 1 × TAE (40 mM Tris-HCl, 20 mM acetic acid, 1 mM EDTA pH 8) for 15 minutes. Bands were visualised on a UV transilluminator, excised, placed in 15 μL of ddH2O and stored at -20°C for sequencing reactions.

### Sequencing

PCR products were purified using a QIAquick PCR Purification Kit (QIAGEN, Germany) as per manufacturers instructions, the DNA quantified with a NanoDrop spectrophotometer (ND-1000 NanoDrop Technologies, USA), and 10–20 ng of product used as a template for direct sequencing reactions. Sequencing reactions were analysed using an ABI automated sequencer (Applied Biosystems, USA) at a commercial facility (Royal Perth Hospital, West Australian Genome Resource Centre). Both DNA strands were sequenced using the original primers and a 10 μl reaction mix containing 2 μl of BigDye Terminator Mix (BDTM) version 3.1 (Applied Biosystems, USA). Chromatographs were checked by eye using the software Chromas Lite version 2.0 and ambiguous parts were removed. The basic local alignment search tool (BLAST) [39] was used to compare sample sequences to other bacterial sequences found on the database NCBI (National Center for Biotechnology Information). Pairwise comparisons were made with EMBL-EBI Emboss Pairwise Alignment Algorithms . Sequences were deposited in the GenBank database under the following accession numbers: EU383035 to EU383052.

## Results

### Abundance of photosynthetic sponges

A total of 63 different sponge species from 7 transects were examined for the presence of photosymbionts. 30 sponge species had high levels of autofluorescence and were categorized as 'positive' for photosymbionts (48%). 10 species had 'low to medium' levels of autofluorescence (16%) and 23 species were 'negative' (36%) (Table 2). Samples with a positive PAM fluorometry result had medium to high cyanobacterial autofluorescence. The observation of sponges with low to medium levels of autofluorescence, and correspondingly low PAM readings, was unexpected. As the nature of these associations is not known, we did not count these sponges as positive or negative for photosynthetic symbioses, but made a separate category for them.

Between 2 and 22 photosynthetic sponge individuals and 2 to 13 photosynthetic sponge species occurred on the transects, and the percent and number of photosynthetic sponge species was higher for the southern sites at Eagle Bay and Busselton Jetty (Table 2). Of the photosynthetic sponges only three species, *Chondrilla australiensis, Chondropsis *sp. and *Mycale *sp., occurred on more than one transect. However, other photosynthetic sponges were observed at multiple sites, but did not occur on more than one transect line. Many sponges could not be identified to known species, and probably represent previously poorly described or new species.

### Types of photosynthetic symbionts in sponges: Molecular and microscopy results

**Figure 1:**
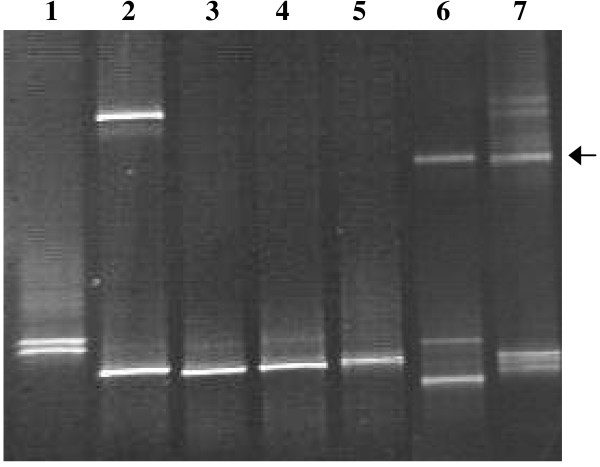
**DGGE of cyanobacterial symbionts**. 16S rDNA Denaturing Gradient Gel Electrophoresis of cyanobacterial symbionts occurring in sponges in this study. 1: Host = *Spongia *sp. Both high and low bands = *Synechocystis *sp., differing by 2 base pairs. 2: Host = *Chondropsis *sp. High band = uncultured phototrophic diatom. Low band = *Oscillatoria spongeliae*. 3: Host = *Psammastra *sp., symbiont = "*Candidatus *Synechococcus spongiarum". 4: Host = *Chondrilla australiensis*, symbiont = "*Candidatus *Synechococcus spongiarum". 5. Host = *Chondropsis *sp., symbiont = *Oscillatoria spongeliae*. 6. Host = *Xestospongia *sp. High band = uncultured sponge bacterium. Medium band not able to be sequenced, appearance matches *Synechocystis*. Low band = chloroplast. 7: Host = *Mycale *sp. 1 High (arrow) and middle bands = *Synechocystis *sp., 96.6% similarity to each other.

Sequences were obtained for the symbionts of 10 photosynthetic sponges. Symbionts from *Crella *sp., a Calcarea species, *Lissodendoryx *sp., *Phyllospongia *sp., *Cymbastela marshae *and *Hippospongia *sp. were sequenced directly using the primers cya359F and cya1459R. The sequencing of symbionts in a number of other sponges was problematic and may have been caused by multiple factors including, in some instances, the presence of more than one type of symbiont. DGGE of amplified 16SrDNA from 7 sponges was performed (Figure 1) where this was suspected. Sequencing of the resulting bands was successful for *Spongia *sp., *Mycale *sp. 1, *Chondropsis *sp., *Xestospongia *sp., and *Psammastra *sp., resulting in sequences less than 450 bp. An overview of these results is given in Table 3.

**Table 3:** Overview of the 16S rDNA sequencing results of sponge symbionts in temperate Western Australia.

Symbionts	Sponges			
*Synechoccus spongiarum *clade	Chondrilla australiensis*	*Hippospongia *sp.	*Phyllospongia *sp.	*Psammastra *sp.
*Synechococcus *species	Calcarea species	*Haliclona *sp.*	*Crella *sp.	
*Synechocystis *species	*Mycale *sp. 1	*Spongia *sp.		
*Oscillatoria spongeliae *clade	*Chondropsis *sp.			
Undescibed cyanobacterium	*Cymbastela marshae*			
Rhodophyte	*Lissodendoryx *sp.			

*These symbionts were sequenced by Usher et al. (2004a).

#### Synechococcus species

The cyanobacterial symbionts of *Crella *sp. (transect WP1) and an unidentified Calcarea species (EB) were identified as two different *Synechococcus *strains with 16S rDNA analysis (Table 3). *Crella *sp. only had medium levels of cyanobacterial autofluorescence and the symbiont sequence resulted in a close GenBank match with the free-living *Synechococcus *strain WH 8016 (99.6% sequence similarity to accession number AY172834). The 16S rDNA sequence of the Calcarea species symbiont resulted in 98.8% sequence similarity to another free-living *Synechococcus *(accession number DQ248009.1). This sponge had high levels of cyanobacterial autofluorescence and a relatively high PAM reading of 6.5 maximal relative ETR (electron transport rate).

**Figure 2:**
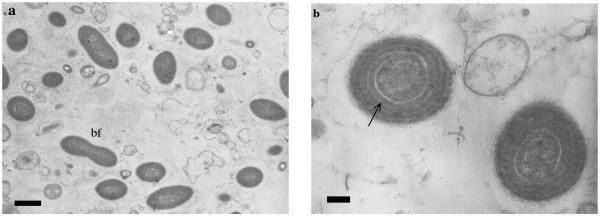
**Symbiont in *Hippospongia *sp**. **a) **TEM analysis for *Hippospongia *sp. showing cyanobacterial symbionts belonging to the "*Candidatus *Synechococcus spongiarum" clade as single cells and during division (bf = binary fission), scale bar = 1 μm. **b) **TEM showing two symbionts with thylakoid spirals (indicated by arrow), scale bar = 200 nm.

Cyanobacterial symbionts belonging to the "*Candidatus *Synechococcus spongiarum" clade were identified in four sponge species: *Hippospongia *sp., *Phyllospongia *sp., *Psammastra *sp. and *Chondrilla australiensis*, based on 16S rDNA sequencing and BLAST searches. All were closely related to existing sequences for this symbiont on GenBank, with similarities over 99%. All four sponge species showed high concentrations of small unicellular coccoid cyanobacteria with fluorescence microscopy. TEM of *S. spongiarum *in *Hippospongia *sp. showed intercellular cyanobacteria about 1 μm long with 4 thylakoid spirals and typical ultrastructure for this symbiont. Some were observed dividing by binary fission (Figure 2a and 2b).

*Synechococcus*-like cyanobacteria were the most common photosynthetic symbionts found in this study. Other sponges that contained medium or high levels of small single-celled cyanobacteria resembling *Synechococcus *by light and fluorescence microscopy included an Aplysinidae species, *Lissodendoryx (Anomodoryx) *sp., Halichondrida (Dictyonellidae?), *Tethya *sp., *Dendrilla *sp., *Haliclona *(*Haliclona*) sp., *Xestospongia *sp. and *Callyspongia *sp. however, these were not sequenced.

#### Oscillatoria spongeliae clade

*Oscillatoria spongeliae *was sequenced from one species of *Chondropsis*, and had a GenBank similarity score of 99.3% to a sponge-associated *Oscillatoria spongeliae *accession number AY615504 [40]. Fluorescence microscopy showed high concentrations of intercellular cyanobacteria, and TEM of *Chondropsis *sp. showed trichomes approximately 4 μm in diameter containing 3 or more cells of variable length (not shown). Their ultrastructure was similar to those in Rützler [6], with domed terminal cells, thylakoids radiating from the centre of cells, and carboxysomes sometimes observed. Phycobiliproteins apparently leak from cells when samples are frozen, causing high background autofluorescence.

#### Synechocystis species

**Figure 3:**
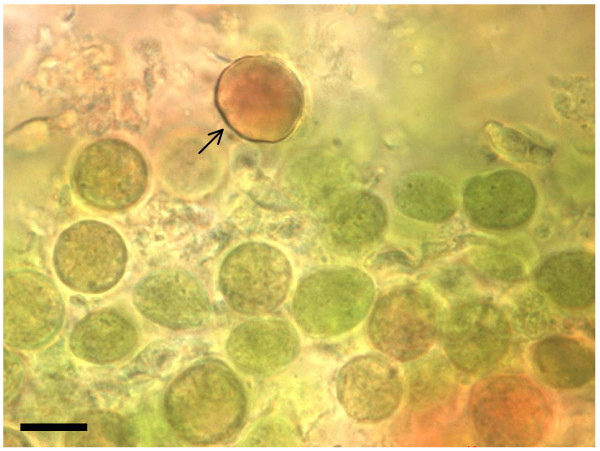
***Synechocystis *sp. Symbionts**. Light microscopy image of two *Synechocystis *species in *Mycale *sp. 1: smaller green coccoid and larger red coccoid symbionts (indicated by arrow), scale bar = 10 μm.

16S rDNA DGGE of *Spongia *sp. symbionts resulted in two bands (Figure 1) that, when sequenced, were only 2 base pairs different. The highest identity score from a BLAST search was 97.4% with the 16S rDNA gene of *Synechocystis trididemni *(accession number AB011380). High numbers of intercellular single-celled cyanobacteria were observed using fluorescence microscopy, and vacuolated, non-spiral thylakoid membranes were observed with TEM (not shown). *Mycale *sp. 1 contained two different *Synechocystis *species that had 94.3% and 96.7% similarity to *Synechocystis trididemni *(accession number AY845229), and had 96.6% similarity to each other. Fluorescence and light microscopy revealed smaller green coccoid cyanobacteria about 10 μm in diameter, and a larger red coccoid symbiont approximately 15 μm in diameter growing in close proximity (Figure 3). Autofluorescence also differed between these symbionts, with the red cyanobacteria emitting orange/red wavelengths and the green cyanobacteria emitting yellow/green wavelengths. High numbers of *Synechocystis*-like cyanobacteria were also observed in *Xestospongia *sp. and *Phorbas *sp. by fluorescence and light microscopy.

#### Unknown Symbionts

*Cymbastela marshae *contained a symbiont with 99.7% 16S rDNA similarity to the symbiont previously found in *Cymbastela marshae *from Fremantle (accession number AY190174; [19]). This symbiont has 98.6% sequence identity to *Oscillatoria rosea *and 97% sequence identity to a *Synechococcus *sp. (accession number AF448076).

DGGE using 16S rDNA resulted in three bands for a *Xestospongia *sp. (Figure 1). One band had 99% sequence similarity to an uncultured sponge bacterium, another closely matched a chloroplast sequence. Sequencing was not successful for the third band. Other types of photosynthetic symbiont may occur, as this study restricted sequencing to representatives of the major groups.

#### Red Algae

**Figure 4:**
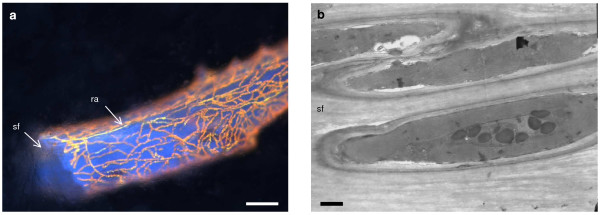
**Rhodophyte symbiont**. **a) **Rhodophyte autofluorescence (ra) in *Mycale *sp. 2 covering spongin fibres (sf), scale bar = 100 μm. **b) **TEM analysis of *Mycale *sp. 2 shows the rhodophytes occur inside spongin fibres (sf), scale bar = 1 μm.

**Figure 5:**
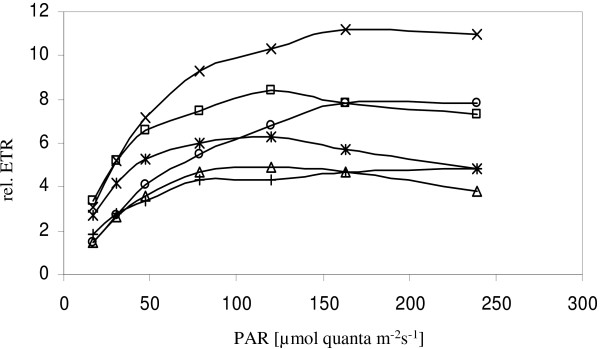
**Light curves**. Recorded light curves using PAM fluorometry: relative photosynthetic electron transport rates (rel. ETR) as a function of irradiance (measured as photosynthetic active radiation PAR) for sponge individuals growing in close proximity on the same transect. □ *Phyllospongia *sp. containing *Candidatus *Synechococcus spongiarum; ○ *Psammastra *sp. containing "*Candidatus *Synechococcus spongiarum"; △ *Spongia *sp. containing *Synechocystis trididemni *like symbionts; × *Cymbastela marshae *containing *Oscillatoria sp*., + *Mycale sp*. 2 containing a Rhodophyte and * unidentified Calcarea species containing a *Synechococcus sp*.

A rhodophyte species was found to be associated with the sponge *Lissodendoryx *sp. The maximum GenBank identity score was 95% for the accession number AY731518, the 16S rDNA gene of *Palmaria palmata *(Rhodophyta). Fluorescence microscopy indicated that similar symbionts also occurred along the spongin fibres of *Crella *sp., *Clathria *sp., *Mycale *sp. 2 (Figure 4a), *Dictyodendrilla *sp. and *Iotrochota *cf. *baculifera*. Sponge skeletons appear red where the densities of rhodophytes are high. TEM observations show the rhodophytes occur inside spongin fibres, splitting them as they grow (Figure 4b), although it is not known if this always occurs. The ultrastructure of the symbionts is similar to *Acrochaetium spongicolum *in *Mycale laxissima *observed by Rützler [6]. Both *Lissodendoryx *sp. and *Mycale *sp. 2 were semi-translucent and encrusting or fan-shaped, however rhodophytes were observed throughout the matrix of several densely pigmented sponges. Two species of *Clathria *sp. and one *Crella *sp. were opaque orange and a *Dictyodendrilla *sp. was opaque grey. *Dictyodendrilla *sp. was the only species harboring rhodophytes without siliceous spicule bundles within the fibres. This sponge had relatively low PAM readings (relative ETR of 2.2) in comparison to *Mycale *sp. 2 (Figure 5).

### PAM fluorometry

Both *Psammastra *sp. and *Phyllospongia *sp. hosted *Synechococcus spongiarum *and occurred in close proximity in the same light conditions. However, the recorded light curves show saturation for *Psammastra *sp. at around 163 μmol quanta m^-2^s^-1 ^with a maximal relative ETR of 7.8 while *Phyllospongia *sp. saturated at 120 μmol quanta m^-2^s^-1 ^with a maximal relative ETR of 8.4 (Figure 5). The light curve for *Cymbastela marshae *(symbiont *Oscillatoria *sp.) showed saturation of photosynthetic electron transport at around 163 μmol quanta m^-2^s^-1 ^and the maximal relative electron transport rate was 11.2. *Spongia *sp. hosted a *Synechocystis trididemni *like symbiont and saturated at 120 μmol quanta m^-2^s^-1 ^with a maximal relative ETR of 4.9. Using molecular techniques a *Synechococcus *sp. was detected in a Calcarea species and the light curve showed saturation at 120 μmol quanta m^-2^s^-1^with a maximal relative ETR of 6.3. A rhodophyte symbiont was found in *Mycale *sp. 2 with saturation at around 240 μmol quanta m^-2^s^-1 ^and a maximal relative ETR of 4.8.

Rapid light curves contain information that enables the determination of quantum yields (Y) of photosystem II and induction and saturation characteristics of photosynthesis, and can therefore be used to compare sponges harboring the same symbionts under different light conditions. However, as the number of measurements for identical sponge species from different transects was too low, this analysis was not conducted. Nonetheless, the method provides an indication of the saturation characteristics of different sponge symbionts that occur close to each other in the same light conditions.

## Discussion

This study is the first to demonstrate that photosynthetic sponges are abundant and diverse in a temperate region in south Western Australia. An average of 48% of species and 63% of individuals were found to be strongly photosynthetic, with an additional 16% of species and 11% of individuals having low to medium levels of photosynthetic symbionts. These percentages are comparable to those found in temperate south east Australia. Roberts and colleagues [29] estimated that over 65% of the sponges from that region may contain photosynthetic symbionts. These percentages are also comparable to those found on tropical reefs. Steindler et al. [27] found an average of 71.5% of sponge species in the Caribbean were photosynthetic, while Erwin and Thacker [41] report that over 30% of sponge species, also in the Caribbean, were photosynthetic. Wilkinson [28] demonstrated an average of 40.2% of sponge species were photosynthetic on the Great Barrier Reef (GBR), Australia, with a similar percentage reported for the Caribbean, and Rützler [6] reported that 45% of sponges on Caribbean reefs hosted small unicellular cyanobacteria. Sponges containing photosynthetic symbionts occurred in 20–75% of sponge diversity reported at Carrie Bow Cay (Belize) [42] on 10 m line transects. The percentages of 48–64% photosynthetic sponges per transect observed in our study are comparable to these data.

Symbionts belonging to the "*Candidatus *Synechococcus spongiarum" clade were the most common cyanobacteria, however, *Synechocystis *species and *Oscillatoria spongeliae *were also found, along with other *Synechococcus *species and a species of red algae (Rhodophyta).

Sponge hosts containing these symbionts sometimes occurred in close proximity to one another, but it is unknown how the symbionts are acquired by their hosts. The mode of symbiont transmission is only known for one sponge species that occurs in WA, *Chondrilla australiensis*, and this species transmits cyanobacterial symbionts vertically from parent to offspring via the eggs, and sometimes the sperm [19]. However, other symbioses may have different modes of transmission and acquisition of symbionts. If cyanobacterial symbionts occur in the water column sponges must be highly selective for symbiont type. Some of these symbioses are likely to be ancient, and their establishment in nutrient-rich coastal regions where free-living *Synechococcus *are particularly common [43] is possibly encouraged via a close, long-term association [44].

### " Candidatus Synechococcus spongiarum"

The "*Candidatus *Synechococcus spongiarum" clade is the largest sponge-specific clade of bacterial symbionts known, and Taylor et al. [7] reported a total of 21 sponge species from which it has been sequenced. Since then Erwin and Thacker [41] have sequenced *S. spongiarum *in a further 9 sponge species. We found three additional sponge species, *Hippospongia *sp., *Phyllospongia *sp. and *Psammastra *sp. with this symbiont in high concentrations, bringing the total number of sponge species known to have this symbiont to 33.

### Synechocystis trididemni

This is the first report of *Synechocystis trididemni *like symbionts from temperate Australia. This symbiont was reported in the sponges *Prianos *aff. *melanos*, *Spirastrella *aff. *decumbens, in *an unidentified sponge on the Great Barrier Reef, Australia [45], and in sponges from Palau [46] and New Zealand [47]. We obtained two 16S rDNA *Synechocystis *sequences from each of the sponges *Spongia *sp. and *Mycale *sp. 1. The symbionts from *Spongia *sp. differed by only 2 base pairs, and had 97.4% and 97.2% sequence identity to *S. trididemni *from the colonial ascidian *Trididemnum solidum*. The two symbionts in *Mycale *sp. 1 were only 96.6% identical to each other, suggesting that there are two different *Synechocystis *species within the same sponge. When viewed with light and fluorescence microscopy two types of symbiont were observed in *Mycale *sp. 1, a smaller green coccoid cyanobacterium about 10 μm in diameter, and a larger red coccoid symbiont approximately 15 μm in diameter. Autofluorescence wavelengths also differed, suggesting differences in phycobiliprotein concentrations, despite their close proximity within the host sponge. These two symbionts possibly have maximum photosynthetic efficiencies at different wavelengths, enabling their host to grow in a wider range of light levels. Two more sponge species, *Xestospongia *sp. and *Phorbas *sp. contained relatively large coccoid cyanobacteria, resembling *Synechocystis*, in high concentrations.

### Oscillatoria spongeliae

The cyanobacterium *Oscillatoria spongeliae *is possibly best known from *Dysidea sp*., but has been described from a number of sponges in different areas of the world, including Palau [40] and Guam [48]. It was observed in *Dysidea *sp. on the Great Barrier Reef, Australia [49,50], but this is the first report of *O. spongeliae *in Western Australia, where it occurs in high densities in the surface tissues of a *Chondropsis *species.

#### Unknown Symbiont

The distinctive "hairy" cyanobacterium in *Cymbastela marshae *has not been observed in other sponges, or reported as free-living, but was again found in this study. Its phylogeny is uncertain, however, it has 98.6% sequence identity to *Oscillatoria rosea*. It occurs in high densities within the sponge and symbioses appear to be stable over time and space, having been found in *Cymbastela marshae *in Fremantle [26], and Busselton, WA (this study), in different years. Taylor and co-workers recently demonstrated a phylogenetic relationship with *Oscillatoria rosea *and the *Synechococcus *sp. strain PCC7002 [7].

#### Rhodophyta

Filamentous rhodophytes with similar gross morphology were observed in at least 7 sponge species in this study, growing through and over spongin fibres and giving them a red colouration. Similar spongin-permeating algae, *Ostreobium *cf. *constrictum *and *Acrochaetium spongicolum*, were described from Belize [6], and the Mediterranean [51]. Rhodophyte/sponge symbioses also occur on the Great Barrier Reef, Australia [52], and in southern Australia [5], however, in these symbioses the algae are considerably larger.

This study is the first to investigate the molecular phylogeny of rhodophyte symbionts in sponges. Our 16S rDNA sequence of this symbiont in *Lissodendoryx *sp. was 95% identical to *Palmaria palmata*, and may be a new species. However, further work is needed to establish if all 7 sponge species host the same species of rhodophyte. Using TEM, their ultrastructure was similar to *Acrochaetium spongicolum *in *Mycale laxissima *observed by Rützler [6]. There were no indications that the rhodophytes harm the host, although this is possible as the algae are spongin-boring in at least some cases. In the *Haliclona cymaeformis/Ceratodictyon spongiosum *symbiosis the rhodophyte partner donates a small amount of photosynthate to the sponge host [53], and may provide support [52]. In return the rhodophyte partner benefits from the provision of nitrogen by the sponge, boosting algal growth rates in comparison to algae not in symbiosis [53]. Neither *Haliclona cymaeformis *or *Ceratodictyon spongiosum *have been observed living without the other partner [52].

Sponges containing rhodophytes included densely pigmented orange and dark grey species, and it is interesting that algae can grow successfully deep in the body of these sponges. It is possible that light is conducted to the algae by spicules, as all the sponge species containing rhodophytes in this study except *Dictyodendrilla *sp. have siliceous spicule bundles contained within the fibres on which the algae grow. Like cyanobacteria, but unlike most other algae, rhodophytes contain phycobiliproteins. These accessory photosynthetic proteins capture a broad range of the light spectrum and enable organisms to grow at much lower light intensities than terrestrial plants [54]. It would therefore seem that, like cyanobacteria, rhodophytes are able to grow in the low light intensities found within the matrix of sponge hosts, and are closely associated with the spongin fibres both for physical support and light transmission via the spicules. The transport of photo-active radiation via spicules was recently shown in a living *Tethya aurantium *[55].

#### Levels of cyanobacterial autofluorescence

It is interesting that different levels of cyanobacterial densities were observed in sponges in this study, ranging from low to high. Sponges with very high densities of cyanobacteria resulted in a strongly positive PAM result, while other sponges with low to medium concentrations of symbionts resulted in low to medium PAM readings. Similar observations have been made by Erwin and Thacker [41], who found low and intermediate chlorophyll *a *levels in sponges in the Caribbean, but the nature of this type of association is unknown. It is possible that sponges in the "low" density group retained free-living cyanobacteria from the water column on which they were feeding, either in choanocytes or other areas of the body, and for this reason they were not included in the group that was positive for photosynthetic symbionts. The cyanobacteria in the "medium" density group may also result from water column feeding, but it is not known if these are retained by the sponge over time i.e. symbionts. The cyanobacteria in one sponge from the "medium" group, a *Crella *sp., were closely related to free-living *Synechococcus *species. This may represent a nascent symbiosis in which the cyanobacterial symbionts and sponge host are negotiating the association, with symbionts being consumed more often than is usually the case. However, it is interesting to note that the unidentified Calcarea species also had symbionts closely related to free-living cyanobacteria but these occurred consistently in this species and at high concentrations.

Sponges with microbial symbionts must have regulatory mechanisms to avoid symbionts overwhelming host tissues. Several of these mechanisms have been proposed including the sponge consuming enough of the products from photosynthesis to restrict cyanobacterial growth, and the sponge eating or ejecting the symbionts when stressed [7,56]. However, we still lack a clear picture of the factors and mechanisms that control these associations. More experimental work is needed to determine sponge-symbiont interactions and regulation.

The Diving PAM was a useful method for determining photosynthetic sponges *in situ*, and provided an indication of the density of photosynthetic symbionts in the sponge tissue.

DGGE using 16S rDNA was useful for separating and sequencing cyanobacterial symbionts where more than one species was present within a host. However, it appears that our gel would have benefited from running longer than the 3 1/2 hours we used, as the bands for "*Candidatus *Synechococcus spongiarum" and *Oscillatoria spongeliae *were not noticeably separated. It is interesting that sponges containing more than one photosynthetic symbiont are relatively common.

#### Occurrence of photosynthetic sponges

There are likely to be a number of factors that influence the occurrence of photosynthetic sponges, including light conditions, sedimentation and possibly nutrient levels. In WA the Leeuwin Current transports nutrient-poor water from the tropics southwards in winter, however, this flows well offshore from our sampling sites. Chlorophyll *a *levels in the water column at inshore locations indicate that nutrient levels remain relatively high all year, but are particularly high in the wet winter months from run-off from land, reaching 1 mg m^-3 ^[34]. During the summer months the Capes Current, which begins in the Cape Leeuwin and Naturaliste region (close to our sampling sites at Busselton Jetty and Eagle Bay), is created from an upwelling of cold, nutrient-rich water [34], potentially influencing nutrient levels at our more southerly sampling sites.

Interestingly, the percentage of photosynthetic sponge species was higher at the Busselton Jetty and Eagle Bay sites than in the Perth metropolitan region. Sediment levels were noticeably higher at the latter sites, potentially affecting the success of photosynthetic sponges. Wilkinson [28] observed higher percentages of phototrophic sponges with increasing distance from land on both the GBR and in the Caribbean, perhaps also as a result of decreasing sediment load at offshore sites.

## Conclusion

The high diversity and abundance of photosynthetic sponges found in this study indicates they are as important in temperate Western Australia as they are in tropical zones. Other temperate areas need to be examined to determine if WA is unusual in this respect, or if this finding can be generalized. It appears that there are three common generalist clades of cyanobacterial symbionts of sponges, "*Candidatus *Synechococcus spongiarum", *Synechocystis trididemni *like symbionts, and *Oscillatoria spongeliae *which occur in a wide range of sponges in a wide range of environmental conditions. Further research is needed to examine the different levels of photosynthetic symbionts and the factors and mechanisms that control these associations.

## Authors' contributions

ML and KU carried out transect work including the collection of sponge samples, PAM measurements *in situ *and microscopy. ML completed the majority of PCR analyses and drafted the manuscript. KU designed the study, carried out the DGGE and TEM analysis, organized the permissions and helped to revise the manuscript. Sponges were identified using classical techniques by JF who also helped to revise the manuscript. FB coordinated collaborations and helped to draft the manuscript. All authors read and approved the final manuscript.
